# Need for the *Integrated Nutrition Pathway for Acute Care* (INPAC): gaps in current nutrition care in five Canadian hospitals

**DOI:** 10.1186/s40795-017-0177-8

**Published:** 2017-07-14

**Authors:** Renata Valaitis, Celia Laur, Heather Keller, Donna Butterworth, Brenda Hotson

**Affiliations:** 1 University of Waterloo, 200 University Ave W., Waterloo, ON N2L3G1 Canada; 2 Schlegel-University of Waterloo Research Institute for Aging, 250 Laurelwood Drive, Waterloo, Canada; 3 Concordia Hospital, 1095 Concordia Ave., Winnipeg, MB R2K 3S8 Canada; 4 Winnipeg Regional Health Authority, GG435 820 Sherbrook St., Winnipeg, MB R3A 1R9 Canada

**Keywords:** Malnutrition, Screening, Assessment, Hospital, Implementation, Audit, Strategies

## Abstract

**Background:**

Malnutrition is common in hospitalized patients and is associated with increased mortality, length of stay, and risk of re-admission. The consensus based Integrated Nutrition Pathway for Acute Care (INPAC) was developed and validated to enhance patients’ nutrition care and improve clinical outcomes. As part of the More-2-Eat project (M2E), five hospitals implemented INPAC activities (e.g. screening) in a single medical unit. The purpose of this paper is to demonstrate the care gaps with respect to INPAC activities on these five units prior to implementation. Results were used as part of a needs assessment on each unit, demonstrating where nutrition care could be improved and tailoring of implementation was required.

**Methods:**

Cross-sectional data was collected by site research associates (RAs) using a standardized audit form once per week for 4 weeks. The audit contents were based on the INPAC algorithm. All medical charts of patients on the study unit on the day of the audit were reviewed to track routine nutrition care activities (e.g. screening). Data was descriptively displayed with REDCap™ and analyzed using R Studio software.

**Results:**

Less than half of patients (249/700, 36%) were screened for malnutrition at admission. Of those screened, 36% (89/246) were at risk for malnutrition yet 36% (32/89) of these patients did not receive a dietitian assessment. Also, 21% (33/157) of patients who were not screened at risk were assessed. At least one barrier to food intake was noted in 85% of patient medical charts, with pain, constipation, nausea or vomiting being the most common. Many of these barriers were addressed through INPAC standard nutrition care strategies that removed the barrier (e.g. 41% were provided medication for nausea). Advanced nutrition care strategies to improve intake were less frequently recorded (39% of patients).

**Conclusion:**

These results highlight the current state of nutrition care and areas for improvement regarding INPAC activities, including nutrition screening, assessment, and standard and advanced nutrition care strategies to promote food intake. The results also provided baseline data to support buy-in for INPAC implementation in each M2E study unit.

**Trial registration:**

Retrospectively registered ClinTrials.gov Identifier: NCT02800304, June 7, 2016.

## Background

It is well established that the prevalence of malnutrition in hospitals is high [1–7], yet little has been done systematically to improve the nutrition care of patients during and post hospitalization [8]. A study conducted by the Canadian Malnutrition Task Force found that 45% of patients on medical or surgical wards who stayed more than 2 days were malnourished on admission [3]. Malnutrition often goes undetected [9], which is concerning as it increases mortality, length of stay, and risk of re-admission, even when considering other important covariates such as age, sex, socioeconomic and disease-related characteristics [3, 10–15]. Malnutrition also increases hospital costs [5, 16, 17] and in Canada, this is by approximately $2000CDN per malnourished patient when compared to a well nourished patient [18]. Research has also shown that many patients experience barriers to food intake, such as interrupted meals, or inability to open packages, leading to deterioration of their nutritional status while in hospital [19–21]. Unidentified malnutrition on admission and iatrogenic or worsening of malnutrition increases length of stay and also affects patients after discharge, including an increased likelihood of readmission [3, 11, 19, 22, 23].

Due to the ongoing high prevalence of malnutrition and the pursuit of patient centered care, there is growing focus on the need to build awareness to make improvements and change the culture of nutrition care in hospital [8, 9, 19, 24]. Currently, prevalence rates at admission remain high and research has shown that the lack of a systematic approach to nutrition care in hospitals may exacerbate the issue [25–27]. Keller et al. discovered that many nutrition care practices in Canadian hospitals (including diagnosis, treatment and monitoring) were haphazard [9]*.* This suggests a need for multi-faceted, systematic approaches to nutrition care and malnutrition management [4, 9, 25, 27, 28].

To address the high prevalence, under-diagnosis and under-treatment of malnutrition in acute care patients, the evidence and consensus based Integrated Nutrition Pathway for Acute Care (INPAC) was developed and face validated [9]. The ultimate goal of the pathway is to enhance patient nutrition care and change practice to ultimately improve clinical outcomes of patients [9]. The algorithm outlines steps for detection, treatment and monitoring of malnutrition and food intake among acute care patients and is based on the concept that nutrition care in hospital is multidisciplinary, with all members of the team, including the patient and family, potentially taking on key roles (i.e. nursing can screen; physicians can institute non-volitional feeding; patients can self report barriers to food intake). INPAC focuses on the following activities: i) nutrition screening on admission using a valid, easy to complete nutrition screening tool, such as the Canadian Nutrition Screening Tool, [CNST] [29]; ii) timely and efficient nutrition assessment using the subjective global assessment (SGA) for patients identified to be at risk [30], iii) standard nutrition care strategies (for all patients) that overcome common barriers, thus promoting food intake, iv) advanced nutrition care strategies (e.g. supplements, snacks) for those with SGA score of mild to moderately malnourished, v) timely comprehensive dietitian assessment and individualized treatments for those severely malnourished, vi) food monitoring to identify need for further nutrition interventions, and vii) connecting/referring malnourished patients to community services as they transition out of hospital.

The More-2-Eat (M2E) project demonstrates how INPAC activities can be implemented in five diverse hospitals (one medical unit/hospital) in four provinces across Canada. The aim of M2E is to monitor how each site implements INPAC, including the steps they take, the resources used etc. M2E details can be found in the protocol [31]. Prior to INPAC implementation, baseline chart audits of INPAC activities were conducted to understand current nutrition practices. This paper summarizes these baseline results of the M2E INPAC audit to identify and compare current nutrition care practices in the five medical units, and thus identify gaps in care for targeting and tailoring of INPAC implementation.

## Methods

The M2E study is a multi-site participatory action research project with a pre-post time series design. The baseline data collected for this analysis was cross-sectional, over a 4-week period. Hospitals were selected based on their willingness to change nutrition care practices as presented through a request for proposal process reviewed by Canadian and international implementation experts. Each site is led by a site champion(s), supported by a local Research Associate(s) (RAs). RAs were hospital staff (dietitians and nurses) seconded to the project for data collection. RAs received in-person training regarding data collection methods based on a detailed study procedures manual [31].

### Data collection

M2E is a mixed methods study with continual collecting of quantitative and qualitative data [31]. A site survey provided information on the hospital and the unit (e.g. staffing ratio, number of beds etc.), which was completed by the champion in consultation with necessary departments. To complete the INPAC audit, the RAs collected inpatient information from individual health/medical records on all patients on the study unit, on a single day, once per week for four consecutive weeks (i.e., all patients on the unit during that 24 h period, charts retrospectively reviewed from admission to audit day). As each hospital has a different system for recording patient information, it was not specified which forms needed to be reviewed for data collection. The only requirement was that the data had to be documented on hospital records on the day of the audit and could not be based on verbal or visual accounts of patient care. Audit data was recorded on hardcopy then inputted into RedCAP™ [32], a secure data management system, where it was sent automatically to the team at the University of Waterloo for analysis.

### INPAC audit form

The purpose of the INPAC audit was to track routine nutrition care activities on the M2E unit for every patient currently occupying a unit bed. Data collection occurred on a single day, capturing current nutrition care activities for both new and longer stay patients on the unit at that time. The INPAC audit contents are based on INPAC activities [9] and specifically: date of admission, year of birth, gender, admitting diagnoses, transfer from another unit including emergency; date and result of nutrition screening; presence of SGA to diagnose malnutrition; nutrition diagnosis based on nutrition assessment completed by a dietitian; any specialized diet therapy e.g. diet prescribed; identified barriers to food intake e.g. needing help with eating; if food intake or weight monitoring had occurred; use of advanced care strategies such as preferred foods, oral nutritional supplements (ONS); and if standard nutrition care strategies had been implemented e.g. medication for nausea. The individual completing the audit recorded if screening and assessment had been completed by staff as part of usual care. The audit form was drafted by the M2E core team (*n* = 4) and reviewed by the larger M2E team, including the champions and RAs. This team of reviewers included dietitians, physicians, nurses, food service managers, hospital management and experts in implementation. The final draft was piloted during the first week of the baseline data collection and minor changes were made based on feedback from the RAs using the tool.

### Analysis

At the end of baseline data collection, INPAC audit data was descriptively analyzed for each site and amalgamated. Baseline data were analyzed using R Studio including descriptive statistics (means, standard deviation, proportions). Comparison across sites was completed with ANOVA and chi square. Due to the large number of comparisons completed, *p* < 0.01 was used to determine statistically significant associations.

### Ethics

Ethical approval was obtained from all participating hospital research ethics boards and the Office of Research Ethics at the University of Waterloo (ORE #20590). Patient consent was not required for the audit, as patient identifiers were not used and there was no interaction with the patient. Some hospital ethics boards required the posting of a notification of this research activity for patients and family/friends to review. Any patient/family requesting to be omitted was not included in the audit. This study was retrospectively registered at ClinTrials.gov, NCT02800304 on June 7, 2016.

## Results

### Site descriptions

Five hospitals from four provinces in Canada were selected to have one unit each included in M2E. The hospital sites selected to participate were diverse in a variety of ways; two were community hospitals, while three were academic. The hospital sizes ranged from 150 to >1000 beds, with the study unit size being relatively consistent with 27-50 beds. The nurse to patient ratio ranged from 1:4-1:9 for day shifts and 1:5-1:11 for night shifts. The reported average length of stay for 2015 was 9.93 (SD: 0.69) days ranging from 5.5-10.9 days. Some study units had specific specializations, including a stroke unit and a respiratory unit. One was also piloting an Accountable Care Unit [33]. Some types of staff also differed across sites regarding their respective roles in nutrition/food/mealtime care. For example, some sites had Dietary Technicians (DT) or Clinical Nutrition Assistants (CNA), while others did not. Health Care Aides (HCAs) were available on some units to help with setting patients up for mealtimes, while others relied solely on regulated nursing staff. At some sites, food service workers delivered the food directly to the patient. At other sites, food services delivered food to the unit and then nursing/support staff delivered the food to patients. Table 1 provides further description on each site and its characteristics.

**Table 1:** Site characteristics

	Site A	Site B	Site C	Site D	Site E
Hospital type	Academic	Academic	Community	Academic	Community
Hospital size (# of beds)	430	1100	150	798	186
Unit size	35 beds	27 beds	50 beds	28 beds	34 beds
Nurse: patient	Days 1:9 Nights 1:6	Days 1: 4 Nights 1: 6	Days 1:5 Nights 1:8	1:5	Day: 1:6 Evening: 1:7 Night: 1: 11
Hospital Reported Average Length of Stay on Study Unit	9.3 days	10.3 days	5.5 days	9.3 days	10.9 days
Unit specializations	Accountable Care Unit	Specialized staff	Acute Stroke	Respiratory	Family Medicine
Nutrition-related Roles Present on Unit	• Diet clerk • RD • Food service workers	• Diet clerk • Diet tech • RD • Food Attendants	• Clinical Nutrition Assistant • Dietary Aide • RD	• RD • Food service workers	• Dietary Aide • Diet Clerk • RD • Food service workers
Food delivery	Tray service by food service worker	Tray service by food attendants	Tray service by dietary aide	Tray service by food service worker	Tray service by dietary aide/health care aide
Involvement in original Canadian Malnutrition Task Force study	Yes	No	Yes	Yes	No
Patient meal day cost	N/A	$30.22	$13.97	$41.04	$19.18
Average bed cost/day	$1035.00	$531.88	$325.00	$1000.00	$327.00

Data reported on site survey

### Patient demographics and diagnosis

During baseline data collection, a total of 700 audits were completed across the five sites (note there were some duplicate patients due to repeat audits in consecutive weeks). Fifty-three percent of audits were based on female patients, and the mean patient age was 71 years (SD =16.26) with ages ranging from 21 to 100 years old. Almost one in five audits were based on patients who were transferred from another unit. There were statistically significant differences among sites regarding average patient age (F = 7.072, *p* = 0.008) and gender (χ^2^ = 17.60, *p* < 0.0001). Oldest patients were at Site A (76 yrs) and C (76 yrs), while Site D had the most males (60%). The most commonly reported admitting diagnoses were respiratory (21%), cardiovascular (12%), infection (12%), neurologic (11%) and gastrointestinal (10%); however, there was variation by site with statistically significant differences between sites for all diagnoses but cancer (χ^2^ = 2.41, *p* = 0.66) (See Table 2). For example, 74% of patients in site D had a respiratory diagnosis, which is expected as this was a respiratory unit, whereas Site B had a higher proportion of patients with an admitting diagnosis of infection (29%).

**Table 2:** Patient demographics & common diagnoses by site (*n* = 700)

	Overall (*N* = 700)	Site A (*n* = 152)	Site B (*n* = 119)	Site C (*n* = 159)	Site D (*n* = 131)	Site E (*n* = 139)
Age (yrs) *Mean (SD)*	71 (SD: 16.26) *	76 (SD: 15.31)	71 (SD: 19.43)	76 (SD: 14.57)	64 (SD: 13.73)	73 (SD: 15.65)
Gender (male)	46.7% ** (*n* = 327/700)	40.1% (*n* = 61/152)	42.9% (*n* = 51/119)	39.6% (*n* = 63/159)	59.5% (*n* = 78/131)	53.2% (*n* = 74/139)
Transferred from Other Unit	19.0%** (*n* = 122/643)^a^	18.4% (*n* = 23/125)^a^	22.7% (*n* = 25/110)^a^	13.1% (*n* = 20/153)^a^	33.3% (*n* = 39/117)^a^	10.9% (*n* = 15/138)^a^
Admitting Diagnosis Categories	All Sites %(n)	Site A % (n)	Site B %(n)	Site C %(n)	Site D %(n)	Site E %(n)
Cardiovascular	11.9 (83) **	5.9 (9)	9.2 (11)	18.9 (30)	3.1 (4)	20.9 (29)
Gastrointestinal	10.1 (71) **	16.4 (25)	10.9 (13)	12.6 (20)	0 (0)	9.4 (13)
Genitourinary	4 (28) **	3.9 (6)	0.8 (1)	9.4 (15)	1.5 (2)	2.9 (4)
Respiratory	21.3 (149) **	10.5 (16)	5.9 (7)	6.3 (10)	74 (97)	13.7 (19)
Musculoskeletal	7.7 (54) **	21.1 (32)	4.2 (5)	6.3 (10)	0 (0)	5.0 (7)
Neurologic	11.4 (80) **	2.0 (3)	15.1 (18)	25.2 (40)	0 (0)	13.7 (19)
Infection	12.1 (85) **	11.8 (18)	29.4 (35)	10.7 (17)	7.6 (10)	3.6 (5)
Cancer	6.7 (47)	8.6 (13)	4.2 (5)	6.9 (11)	7.6 (10)	5.8 (8)
Other	8.4 (59) **	8.6 (13)	7.6 (9)	0 (0)	3.1 (4)	23.7 (33)

*indicates statistically significant difference across sites (*p* < 0.01) ** (*p* < 0.0001)

^a^ Indicates missing data

*n.s* not statistically significant

### Nutrition care practices

#### Nutrition screening

Three of the five sites already had screening for nutrition risk in place, using the CNST [29], the Malnutrition Screening Tool (MST) [34] or a site-specific screening form. The remaining two sites had a small proportion of screening completed by the M2E RA for the purpose of data collection during the baseline period; these participants were not included in the counts provided below and in tables. In total, screening was completed on 36% (*n* = 249/700) of patients, ranging from 0% to 76%, with statistically significant differences among sites. Of those screened, 36% (*n* = 89/246; three patients missing as status not recorded) of patients were considered ‘at-risk’ for malnutrition. There were no statistically significant differences in risk status by the three sites that completed screening (Table 3).

**Table 3:** Estimates of screening; nutrition assessment; nutrition diagnoses; food intake & body weight monitoring by site (*n* = 700)

Screening & risk identification:	Overall (*N* = 700)	Site A (*N* = 152)	Site B (*N* = 119)	Site C (*N* = 159)	Site D (*N* = 131)	Site E (*N* = 139)
Screened for malnutrition	35.5% ** (*n* = 249/ 700)	76.3% (*n* = 116/ 152)	0% (*n* = 0/119)	25.8% (*n* = 41/159)	0% (*n* = 0/131)	66.1% (*n* = 92/139)
Of those screened, AT RISK	36.1% (*n* = 89/246)^a,b^	31% (*n* = 35/113) ^*a*^	N/A	56.1% (*n* = 23/41)	N/A	33.7% (*n* = 31/92)
Comprehensive dietitian nutrition assessment						
% receiving comprehensive dietitian assessments	27.9% ** (*n* = 195/700)	25% (*n* = 38/152)	16.8% (*n* = 20/119)	23.9% (*n* = 38/159)	38.9% (*n* = 51/131)	34.5% (*n* = 48/139)
Nutrition diagnoses						
% who received a nutrition diagnosis	26.1% ** (*n* = 183/700)	24.3% (*n* = 37/152)	13.4% (*n* = 16/119)	22.0% (*n* = 35/159)	37.4% (*n* = 49/131)	33.1% (*n* = 46/139)
Food intake monitoring						
% who had food intake monitored	6.2% ** (*n* = 43/699) ^*a*^	0%	4.2% (*n* = 5/119)	0.6% (*n* = 1/158) ^*a*^	8.4% (*n* = 11/131)	18.7% (*n* = 26/139)
Body weight recorded at admission						
% who had their body weight recorded at admission	47.9% ** (*n* = 335/700)	14.5% (*n* = 22/152)	16.8% (*n* = 20/119)	78% (*n* = 124/159)	93.1% (*n* = 122/131)	33.8% (*n* = 47/139)
Body weight monitoring						
% who had their body weight monitored during their time in hospital	17.5% ** (*n* = 122/699) ^*a*^	17.8% (*n* = 27/152)	10.9% (*n* = 13/119)	1.9% (*n* = 3/158) ^*a*^	16% (*n* = 21/131)	41.7% (*n* = 58/139)

**indicates statistically significant difference across sites (*p* < 0.0001)

^a^Indicates missing data

^b^Indicates use of Fishers’ exact test rather than chi-square

#### Nutrition assessment

Malnutrition assessment using the subjective global assessment (SGA) was not routinely completed at baseline. Twenty-eight percent of all patients (*n* = 195/700) across the five sites had a comprehensive dietitian nutrition assessment completed (Table 3). Of the 89 patients found to be at risk through nutrition screening, 32 (36%) did not have a dietitian assessment completed (Table 4). Of those who were screened and found to be *not* at risk (*n* = 157), 33 (21%) of patients received a dietitian assessment.

**Table 4:** ‘At-Risk’ vs. ‘No Risk’ patients receiving comprehensive Dietitian Assessments

	Assessed by RD	Not assessed by RD	Total
At Risk	57 ^*a*^	32 ^*a*^	89 at risk
Not At Risk	33	124	157 not at risk
Total	90 assessed	156 not assessed	246 patients

102 additional patients who were not screened received dietitian assessments – they are not included in this chart

^a^ Indicates missing data

Twenty-six percent of patients were assessed to have a specific nutrition diagnosis: 42% had inadequate oral intake; 25% had ‘other’ nutrition diagnoses (i.e. altered gastrointestinal [GI] function, altered nutrition related laboratory values, inadequate enteral nutrition infusion); 18% had unintended weight loss; and 13% had a swallowing difficulty. Only 3% had malnutrition as an identified nutrition diagnosis.

#### Standard and advanced nutrition care strategies

The majority of patients (83%) received standard nutrition care strategies, many of which addressed the most commonly reported barriers to food intake as identified below. For example, across the sites 69, 54, and 41% of patients’ received medications to address pain control, constipation/diarrhea, and nausea respectively, and thus promote food intake. Other standard nutrition care strategies reported included assistance with positioning during meals (19%), and dysphagia diagnosed and addressed with diet (17%). Significant site differences were seen in the proportion of patients within a site who received various care strategies (Table 5).

**Table 5:** Standard nutrition care strategies by site (*n* = 700)

Standard nutrition care strategies	Overall (*N* = 700) % (*n*)	Site A (*N* = 152) % (*n*)	Site B (*N* = 119) % (*n*)	Site C (*N* = 159) % (*n*)	Site D (*N* = 131) % (*n*)	Site E (*N* = 139) % (*n*)
% that received any standard nutrition care strategies	83 (581) **	88.8 (135)	74.7 (89)	71.6 (114)	87.7 (115)	92 (128)
*Most commonly reported standard nutrition care strategies:*	*(n = 581)*	*(n = 135)*	*(n = 89)*	*(n = 114)*	*(n = 115)*	*(n = 128)*
Positioning needs for eating addressed	18.9 (110) **	2.9 (4)	42.6 (38)	29.8 (34)	16.5 (19)	11.7 (15)
Eating assistance needs addressed	20.3 (118) **	8.1 (11)	24.7 (22)	37.7 (43)	10.4 (12)	23.4 (30)
Pain control addressed	69 (401) **	84.4 (114)	43.8 (39)	34.2 (39)	92.1 (106)	80.4 (103)
Constipation/diarrhea addressed	54 (314) **	74.8 (101)	48.3 (43)	21 (24)	67.8 (78)	53.1 (68)
Nausea addressed	41.3 (240) **	71.1 (96)	23.5 (21)	13.1 (15)	51.3 (59)	38.2 (49)
Dysphagia diagnosed	17.2 (100) **	2.9 (4)	3.3 (3)	14 (16)	33 (38)	30.4 (39)
Dysphagia addressed with diet	17 (99) **	4.4 (6)	7.8 (7)	11.4 (13)	29.5 (34)	30.4 (39)

More than one strategy may be reported per audit

**indicates statistically significant difference across sites (*p* < 0.0001)

Advanced nutrition care strategies were documented for 39% of patients, with the most common strategies being: providing preferred foods (49%), high energy/protein drinks/supplements (41%), and nutrient dense diets (21%). Sites were using small amounts (e.g. 60 mls) of nutrient dense ONS with medication delivery (e.g. Med Pass) infrequently (6%). Variations were seen among sites in the proportion of patients receiving advanced care strategies (χ^2^ = 135.68, *p* < 0.0001) with a range from 13% (Site C) to 78% (Site E) of patients. Use of preferred foods as a nutrition care strategy was most common in two of the hospitals (site D and Site E). The least commonly reported strategy was to liberalize the diet, with no difference across the sites (*p* = 0.04) (Table 6).

**Table 6:** Advanced Nutrition Care Strategies by Site (*n* = 700)

	Overall (*N* = 700) % (*n*)	Site A (*N* = 152) % (*n*)	Site B (*N* = 119) % (*n*)	Site C (*N* = 159) % (*n*)	Site D (*N* = 131) % (*n*)	Site E (*N* = 139) % (*n*)
Advanced nutrition care strategies						
% that received any advanced nutrition care strategies	39.4 (276) **	42.1 (64)	31.9 (38)	13.2 (21)	34.4 (45)	77.7 (108)
*Types of advanced nutrition care strategies:*	*(n = 276)*	*(n = 64)*	*(n = 38)*	*(n = 21)*	*(n = 45)*	*(n = 108)*
Nutrient dense diet	20.6 (57) **	7.8 (5)	18.4 (7)	0 (0)	24.4 (11)	31.4 (34)
Liberalized diet	3.2 (9) ^a^	0 (0)	2.6 (1)	0 (0)	0 (0)	7.4 (8)
Preferred foods	48.5 (134) **	17.1 (11)	5.2 (2)	4.7 (1)	53.3 (24)	88.8 (96)
High energy/protein milkshakes/drinks/ONS	40.9 (113) **	37.5 (24)	60.5 (23)	28.5 (6)	71.1 (32)	25.9 (28)
Med pass for ONS	6.1 (17) **	0 (0)	21 (8)	33.3 (7)	4.4 (2)	0 (0)
Snacks between meals	11.9 (33) **	4.6 (3)	39.4 (15)	4.7 (1)	26.6 (12)	1.8 (2)
Other (i.e. minced, fluid diets, specialized diets, vitamin supplements)	22.8 (63) **	75 (48)	7.8 (3)	42.8 (9)	4.4 (2)	0.01 (1)

**indicates statistically significant difference across sites (*p* < 0.0001)

^a^Indicates use of Fishers’ exact test rather than chi-square

#### Specialized nutrition care

INPAC audits also tracked if patients received any diet prescriptions or nutrition support beyond a regular diet. More than two-thirds had some form of diet prescription beyond the regular diet. Of those who received some form of specialized nutrition treatment, 19% received non-volitional feeding of which the majority was enteral [82% enteral, 19% parenteral, and 19% supplemental support (in addition to oral intake)]; almost half of those receiving nutrition support were from Site C. There were no significant differences between sites in terms of the proportion receiving non-volitional feeding as enteral support, however, there was variation in terms of parenteral (χ^2^ = 13.8, *p* < 0.001) and supplemental support (χ^2^ = 24.1, *p* < 0.001). A high proportion (71%) of Site A patients receiving non-volitional feeding received parenteral nutrition specifically and patients were more likely to receive more than one form of support at this site (Table 7).

**Table 7:** Specialized nutrition treatment/nutrition support by site (*n* = 700)

Specialized nutrition treatment	Overall (*N* = 700) % (*n*)	Site A (*N* = 152) % (*n*)	Site B (*N* = 119) % (*n*)	Site C (*N* = 159) % (*n*)	Site D (*N* = 131) % (*n*)	Site E (*N* = 139) % (*n*)
% who received any diet prescription beyond the regular diet	68.4 (479)**	59.9 (91)	52.9 (63)	70.4 (112)	77.1 (101)	80.6 (112)
*Nutrition support and type provided*	*(n = 93)*	*(n = 7)*	*(n = 8)*	*(n = 49)*	*(n = 9)*	*(n = 20)*
Enteral	81.7 (76)^a^	57.1 (4)	100 (8)	77.6 (38)	66.7 (6)	100 (20)
Parenteral	19.4 (18)**	71.4 (5)	0 (0)	22.4 (11)	22.2 (2)	0 (0)
Supplemental	19.4 (18)**	42.9 (3)	75 (6)	2 (1)	11.1 (1)	35 (7)

Patients could have received multiple types of nutrition support

**indicates statistically significant difference across sites (*p* < 0.0001)

^a^Indicates use of Fishers’ exact test rather than chi-square

#### Barriers to food intake

Eighty-five percent of INPAC audits noted at least one barrier to food intake while in hospital. The most common barriers identified included: pain (59%), constipation (39%), nausea/vomiting (33%), swallowing difficulty (23%), and cognitive impairment (22%). There were statistically significant differences between sites in terms of proportion of patients with identified barriers to food intake (χ^2^ = 24.6, *p* < 0.0001). Pain was frequently identified in sites D and E. Site E also reported reduced appetite as a common barrier (Table 8).

**Table 8:** Barriers to Food Intake by Site (*n* = 700)

Barriers to food intake	Overall (*N* = 700) % (*n*)	Site A (*N* = 152) % (*n*)	Site B (*N* = 119) % (*n*)	Site C (*N* = 159) % (*n*)	Site D (*N* = 131) % (*n*)	Site E (*N* = 139) % (*n*)
% of audits with barriers identified	84.6 (592)**	79.6 (121)	84.9 (101)	76.1 (121)	90.1 (118)	94.2 (131)
*Most common barriers identified*	*(n = 592)* *% (n)*	*(n = 121)* *% (n)*	*(n = 101)* *% (n)*	*(n = 121)* *% (n)*	*(n = 118)* *% (n)*	*(n = 131)* *% (n)*
Needs positioning	17.7 (105)**	9.9 (12)	13.9 (14)	28.9 (35)	18.6 (22)	16.8 (22)
Needs meal set-up	21.6 (128)**	19 (23)	24.8 (25)	30.6 (37)	22 (26)	13 (17)
Requires eating assistance	15.7 (93)**	9.9 (12)	9.9 (10)	29.8 (36)	1.7 (2)	25.2 (33)
Reduced appetite	17.4 (103)**	10.7 (13)	3.0 (3)	17.4 (21)	11 (13)	40.5 (53)
Cognitive impairment	22 (130)**	25.6 (31)	5.9 (6)	40.5 (49)	3.5 (4)	30.5 (40)
Swallowing difficulty	22.5 (133)**	9.1 (11)	7.9 (8)	18.2 (22)	39.8 (47)	34.4 (45)
Constipation	38.8 (227)**	38.8 (47)	39.6 (40)	12.4 (15)	48.3 (57)	51.9 (68)
Nausea/vomiting	33.4 (198)**	35.5 (43)	29.7 (30)	15.7 (19)	46.6 (55)	38.9 (51)
Pain	58.8 (348)**	45.5 (55)	42.6 (43)	38.8 (47)	68.3 (98)	80.2 (105)

More than one barrier may have been identified per audit

**indicates statistically significant difference across sites (*p* < 0.0001)

#### Monitoring body weight

Forty-eight percent of patients had their body weight recorded at admission (recording within 3 days of admission qualified as an admission weight), although it is unclear whether this was measured, reported or estimated. Only 18% of patients had their body weight *monitored* by being weighed at least once after admission. There were statistically significant differences between sites for admission body weight documentation (χ^2^ = 290.28, *p* < 0.0001) and monitoring (χ^2^ = 87.09, *p* < 0.0001) (Table 3).

#### Monitoring food intake

Only 6% of patients had their food intake monitored during their time in hospital. Food monitoring at baseline would typically be captured by nursing staff on the patients’ chart or as a calorie count ordered by the dietitian or physician. To be counted as monitoring, it had to result in an action if intake was poor. There was a significant difference across sites (χ^2^ = 58.16, *p* < 0.0001). Two of the sites (Site A and C) had no food intake monitoring in place and Site E recorded monitoring most frequently (19%), potentially due to differences in patients and their length of stay (Table 3).

## Discussion

In line with other literature, these results indicate that there is a gap in current and best nutrition care practice in Canadian hospitals [9, 24, 27, 35, 36]. A systematic best practice pathway, such as INPAC, is needed to help identify those patients that might need additional nutrition intervention and ensure dietitians are seeing the patients most in need of their specialized services [9]. Research has shown that hospital staff recognize the need to create effective systems to ensure quality nutrition care, yet it is challenging to put this into regular practice [37, 38].

Results also indicate that there are many statistically significant differences between M2E sites in terms of patient demographics, diagnoses and nutrition care practices. This emphasizes the importance of hospitals completing a needs assessment to determine the most important nutrition care areas to address in their setting. It also highlights the need for the intervention to be adaptable to each individual setting and tailored to meet local priorities. Research has shown that flexible and adaptable interventions have better uptake and increased chances for success [39]. In terms of screening (usually completed by nursing staff or diet clerks), less than half of patients were screened for malnutrition risk at admission. This is consistent with other research indicating that nutrition screening rates are generally poor, ranging from 42% of patients [36] to 64% [10]. Three of the five hospital sites were routinely screening, with only two having relatively good completion rates (76 and 66% of patients) and using validated tools. Many barriers to screening have been identified in the literature, including lack of time, low priority among staff, uncertainty in using existing tools, and not having a simple screening tool embedded into routine practice [38, 40]. The third site (Site C), with a low coverage of screening at 26% of patients, required a long, cumbersome nutrition screening form, which likely affected their success rate with this care activity. Even though screening was in place in these three hospitals, a positive screen only lead to a dietitian assessment in 64% of patients identified to be at risk, raising the question of purpose and ethics of screening if it is not connected to a diagnosis to confirm malnutrition [41]. Screening initiatives need to be coupled with rapid diagnosis, and as suggested by INPAC, this can be facilitated with SGA.

Further, malnutrition was an uncommon nutrition diagnosis when a nutrition assessment was completed yet diagnoses of weight loss and poor intake were common. This may suggest a hesitancy by dietitians to diagnose malnutrition in the Canadian health care system, and potentially the incorporation of SGA into their practice will have a positive impact. Of interest was the high proportion of specialized nutrition care in the form of prescribed diets beyond the regular diet. As dietitians completed a comprehensive assessment in 28% of cases, which may have lead to a diet prescription, this suggests that diet is routinely manipulated by physicians, nurses and Speech Language Pathologists (SLP) and there is an opportunity for further dietitian involvement with some of the more restrictive diet prescriptions.

Recording patients’ body weight at admission (48%) appears to be a more common practice compared to monitoring body weight (18%) during patients’ hospital stay. Results from the Canadian 2010-2011 nutrition Day survey found similar rates of body weights being recorded on admission, although the numbers are increasing (52% in 2010, 67% in 2011) [42]. It is also unclear if the weights recorded at admission in this baseline sample were actual, estimated, or reported, as this was not always indicated in the documentation. The potential variability between these weights can have a significant effect on recommendations for a variety of specialists including pharmacists who prescribe medication based on body weight, occupational therapists who need weights for ordering assistive devices, as well as others. Additionally, research shows that emphasis also needs to be placed on monitoring body weight throughout hospital stay. Collins and colleagues [43] found that nutrition classification declined for 10.3% of hospital patients during their stay. Yet, the Nutrition Care Day Survey indicated that heights and weights were not routinely measured nor were patients monitored for further weight loss during hospital stay [10]. Results from this study also indicate that more attention needs to be placed not only on getting measured weights at admission, but also monitoring weight change over time, particularly in longer stay patients. The INPAC highlights the importance of both care activities.

Research has shown that having food intake of 50% or less during the first week of the hospital admission is significantly associated with a longer length of stay even when adjusting for other important covariates including nutritional status [3]. Low food intake appears to be common in hospital patients, where 57% of Canadian patients consumed less than half of their meal on the day of the nutritionDay survey [42]. This is an important consideration as few M2E sites were monitoring food intake and using this data to change interventions. Challenges in identification of low food intake by hospital staff may contribute to poor rates of monitoring and hence inaction to improve food intake. By increasing food intake monitoring amongst staff, it could lead to supportive actions to improve food intake. Using a process to quickly and accurately identify how much is consumed and have low intake (50% or less) trigger an action is recommended within INPAC [9]. The My Meal Intake Tool (MMIT), completed by patients may be a strategy to support monitoring of food intake [44].

Understanding patient-reported barriers to food intake is necessary to address low food intake in hospital. Many barriers to food intake were identified by staff in all sites, which corroborate findings from other research [19, 23]. The Nutrition Care in Canadian Hospitals (NCCH) study identified numerous barriers in the areas of illness (poor appetite, nausea, pain), eating difficulties (difficulty opening packages, inability to reach meal trays) and organizational barriers (not receiving help to eat, missing meals due to tests) [19]. Barriers identified by the sites in this study were most commonly medically based (e.g. nausea) indicating that other barriers identified in the NCCH study were either not as readily recognized by staff and/or were less frequently addressed or that these barriers were not issues for these sites. The Mealtime Audit Tool (MAT) standardizes the process of monitoring intake and addressing barriers, ultimately improving nutrition care [21].

Standard nutrition care strategies were common among the 5 sites and there appears to be good alignment between care strategies to address the most commonly reported barriers to food intake. For instance, pain is the top reported barrier while pain control was the most common standard nutrition care strategy. However, baseline results also suggest that advanced nutrition care strategies were under-utilized. For example, use of small amounts (e.g. 60 mls) of nutrient dense ONS with medication delivery was low compared to other strategies such as specialized diets and/or supplements given during or between meals. Supplements provided tend to be high-energy, high protein, milkshakes or ONS, however, there is a concern about waste which could be minimized with increased use of the small amount of supplement [45]. This highlights an area for practice improvement.

### Limitations and strengths

This retrospective audit data only provides a snapshot of what was documented regarding current nutrition care practice in the 5 M2E sites during a patient’s stay. Analyses were not completed to show fidelity of nutrition care activities (e.g. screening leading to an assessment), as this was not the purpose. Subsequent analyses during implementation in M2E will address these questions. Barriers that were not formally assessed and strategies that were used either on a sporadic basis or very routinely by staff were likely not documented. For example, pain is mentioned as the largest barrier to food intake, and the most addressed solution, delivery of medication, is more likely to appear in the chart as a medical intervention. However, another barrier to intake is not being able to open food packages, yet without formalized assessment and documentation, this barrier may not have been noted and/or documented. In a busy acute care environment, lack of documentation of standard nutrition care barriers and strategies is likely and thus could be under-reported.

RAs at each site were responsible for local data collection. This approach of having several people collecting data, though necessary, may have led to inconsistencies. External assessors were not feasible for the M2E project overall, due to the nature of the time series design. RAs were based at the local hospital and thus familiar with the charts and location of relevant information. Although each site chose a standard day of the week for INPAC audits to promote comparability over time, across sites, data was collected on different days, including the weekend. These differences allowed for increased variation when considering overall proportion of activities across all sites. As well, there is the potential for individual patients to be represented in more than one audit, due to weekly completion of the audits during baseline; thus prevalence of care activities may be inflated by this double counting. This was unavoidable due to the nature of data collection and the ethics requirement to not include unique identifiers. Finally, the sites included in M2E cannot be considered representative of all hospitals in Canada. The sites applied to be part of the M2E project and needed to demonstrate they were ready to improve their nutrition care practices. Further, sites were chosen based on their current screening practices.

## Conclusion

Results from INPAC audits provide a snapshot of the nutrition care that is being provided on a single medical unit in five Canadian hospitals. Although this sample is not representative of the Canadian context, it provides for the first time, estimates on nutrition care activities beyond screening, which only occurred in ~35% of all patients. Identified nutrition risk was not always followed by an assessment and the proportion of patients seeing a dietitian was relatively low considering the documented level of malnutrition in hospital. Malnutrition was rarely chosen as a nutrition diagnosis, and appears to be underrepresented considering the prevalence of risk. Low rates of food intake monitoring that led to a change in intervention among sites identifies an additional gap in nutrition care. The high use of prescribed diets suggests a need for nutrition care practices that require better coordination with nutrition professionals. Audits also identified strengths of current nutrition care, such as the consideration of key barriers and their follow-through with strategies. Food appears to be the focus for much of the advanced nutrition care strategies provided in these medical units. INPAC audits have provided a strong foundation for making change within the M2E sites. Similar needs assessment is recommended for others choosing to embark on quality improvements to nutrition care, as local data are powerful to stimulating change [46].

## Abbreviations

CNA: Clinical Nutrition Assistants
CNST: Canadian Nutrition Screening Tool
DT: Dietary Technicians
HCA: Health Care Aides
INPAC: Integrated Nutrition Pathway for Acute Care
M2E: More-2-Eat
MAT: Mealtime Audit Tool
MMIT: My Meal Intake Tool
MST: Malnutrition Screening Tool
ONS: Oral Nutritional Supplements
RA: Research Associates
RD: Registered Dietitian
SGA: Subjective Global Assessment
SLP: Speech Language Pathologist
